# Respite care models and practices for persons with intellectual disability: A scoping review

**DOI:** 10.4102/ajod.v12i0.1115

**Published:** 2023-07-25

**Authors:** Toni Abrahams, Sharon Kleintjes

**Affiliations:** 1 Department of Psychiatry and Mental Health, Faculty of Health Sciences, University of Cape Town, Cape Town, South Africa; 2 Western Cape Department of Health, Lentegeur Psychiatric Hospital, Cape Town, South Africa; 3 Western Cape Department of Health and Wellness, Cape Town, South Africa

**Keywords:** intellectual disability, respite care, short break, support, family, services, culture, LMIC

## Abstract

**Background:**

Families are the primary caregivers for persons with intellectual disability (ID), offering informal support to ensure community living. Ensuring families are adequately supported is key to reduce the financial, physical, mental and social toll which long-standing inadequately supported care giving may evoke. Respite care is such a support service offered to caregivers and care-recipients with ID.

**Objective:**

Part of a larger study aimed at developing a respite care service framework for persons with ID for South Africa, the review aimed to elucidate what principles and practices inform current respite care services for this population globally.

**Method:**

The Joanna Briggs Institute (JBI) scoping review framework guided the review. Databases were searched using key and surrogate terms for relevant literature published from 2006 to 2021.

**Results:**

Thirty-one sources met the inclusion criteria from 417 screened sources of evidence. These were published between 2006 and 2020, and included grey and peer-reviewed articles, the latter mostly mixed design. Information on respite care service characteristics, principles, practices, guidelines, evaluations and impacts were found for high- but not low-and-middle-income countries (LMICs).

**Conclusion:**

There is an existing knowledge base that can be drawn on to inform the development of quality respite care. The lack of published information on respite care in LMICs necessitates further research to ensure contextually appropriate respite care developments in these settings.

**Contribution:**

This study contributes to the knowledge base on respite care for persons with ID and points out the research gap in LMICs.

## Introduction

Intellectual disability (ID) is understood as significant global impairment in intellectual and adaptive functioning presenting in early development (APA 2013). It is more prevalent in low- and middle-income countries (LMICs) (Maulik et al. 2011) and is a major disability grouping in Africa (McKenzie, McConkey & Adnams 2013). Families are usually primary caregivers for persons with ID, offering informal or unpaid support (WHO 2011), so ensuring that they are supported is important (Aldersey, Turnball & Turnball 2016). With inadequate support, caring can take an emotional, financial, physical, mental and social toll on caregivers (Neely-Barnes & Dia 2008; Sandy, Kgole & Mavundla 2013; Yantzi, Rosenberg & McKeever 2006), particularly for women who usually carry the burden of care (McKenzie et al. 2013). The toll can affect care-recipients with ID who may experience neglect and abuse (Reid, Sholl & Gore 2013; Strunk 2010).

Respite care is defined as any service affording temporary relief to caregivers to preserve caregiving roles (Chan et al. 2012), and accrues benefits to the person with ID (Guerin et al. 2021), caregivers and family (Whitmore 2017). As an important component of disability support services, respite care models vary by location, provider, duration, frequency, setting, funding, choice and other supports offered, with different contexts requiring different models to offer appropriate services (WHO 2011). Good quality respite care offers benefits such as sustained caregiving roles; improved mental health, physical health, coping, finances, family quality of life and relationships; reduced stress and decreased abuse and institutionalisation (Masulani-Mwale et al. 2016; Reid et al. 2013).

Article 28 of the Convention on the Rights of Persons with Disabilities (CRPD) obligates states to assist with respite care (UN 2006); however, provision remains inadequate especially in LMICs (WHO 2011). A scoping review is useful for identifying key characteristics related to topics of interest (Peters et al. 2020). This review was conducted to identify global models, practices and principles that can inform respite care service development for persons with ID. The scoping review is part of a larger study aimed at developing a service framework for respite care for persons with ID for South Africa.

## Research methods and design

The Joanna Briggs Institute Scoping Review Framework (Peters et al. 2020) guided the review process. The Preferred Reporting Items for Systematic Reviews and Meta-Analyses extension for Scoping Reviews (PRISMA-ScR) (Tricco et al. 2018) guided reporting. The Population, Concept and Context framework was utilised to refine the review questions and inform the search strategy. The population included children and adults with ID; the concepts were models, standards, norms, best practice, guidelines and service frameworks for respite care within the global context. The review questions were: What service models for respite care for children and adults with ID are used globally? What standards, norms, best practice, guidelines and service delivery frameworks inform such services? What service evaluations are performed? What impacts are seen? A protocol was developed but not registered because of the short timeframe available within which to conduct the review. Protocol databases were searched to avoid review duplication. Covidence was used as the screening and data extraction tool.

### Eligibility criteria

Inclusion criteria used to select the articles were: (1) referred to respite care and (2) referred to ID (or surrogate terms), (3) peer reviewed and selected grey literature published between 01 January 2006 (coinciding with CRPD adoption) and 31 December 2021, (4) provided details of the respite care service model and (5) provided detail on respite care service standards, norms, best practice, guidelines, service delivery frameworks or standards of care, and (6) English publications.

### Search strategy

An initial search strategy was developed by the first author, then refined with the faculty librarian for each database. The final search on 21 February 2021 was limited by date and language. The search string for Pubmed, for example, was ‘(intellectual disability or mental retardation or development disability or Intellectual Development Disorder) or (intellectual Disability [Medical Subject Headings {Mesh}]) and (respite or short break or short-term break or short-term relief or relief care or outreach care) or (respite care [MeSH]) and (model or standard or norm or best practice or guideline or framework) or (standard of care [MeSH]) Filters: from 2006 to 2021’.

### Selection of sources of evidence

**FIGURE 1:**
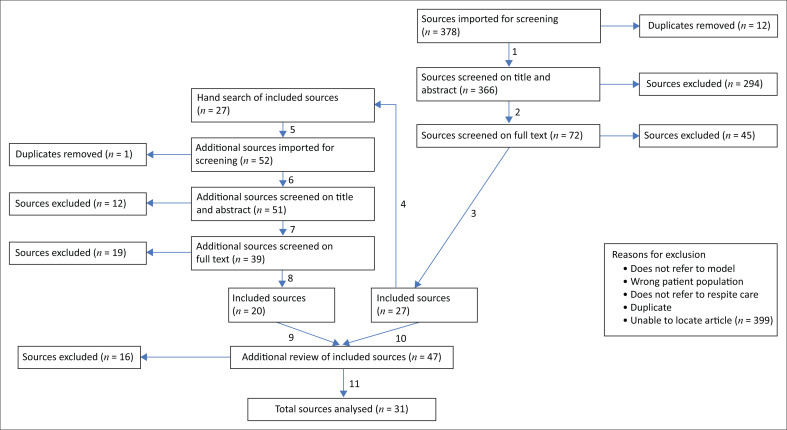
Flow diagram for the scoping review process.

The following databases were searched individually or via EBSCOHost: Academic Search Premier, Africa-wide Info, CINAHL, SocINDEX with Full Text, APA PsycInfo, APA PsycArticles, Healthsource: Nursing/Academic Edition, Scopus, Pubmed, Cochrane and Web of Science. Primo and Google Scholar were searched for grey literature (filtered on conference proceedings and unpublished theses). The results were imported into Endnote. Two reviewers, the first author and a co-reviewer, independently screened initial sources (*n* = 366) on title and abstract, then on full text (*n* = 72), then hand-searched the references of the included sources (*n* = 27) and repeated the screen on the additional source set (*n* = 51) to arrive at an initial inclusion list of 47 articles. Screening conflicts were discussed by the reviewers to come to consensus about inclusion or exclusion at each stage. Although not required, for additional rigour as the reviewers were now fully familiar with the source content of the full set of included articles, an additional full text screen on the list of included sources was performed (*n* = 47), resulting in further sources being excluded (*n* = 16). The reason for exclusion at this point was that the sources were deemed to not sufficiently provide information on standards, norms, best practices, guidelines and service delivery frameworks to warrant inclusion in the final list (*n* = 31). Figure 1 details the process, including reasons for exclusion used across the process. No critical appraisal or risk of bias assessment was conducted as these are not required for scoping reviews (Munn et al. 2018).

### Data charting process

A data extraction template was developed based on an initial overview of the literature on respite care for persons with ID (Peters et al. 2020). The template included fields for characteristics of the included evidence, characteristics of the respite care services and users, details of the standards, norms, best practice, guidelines, principles and frameworks, and details on evaluation and impacts. The initial template was piloted on four articles.

### Synthesis of results

Scoping reviews present and describe identified data, rather than synthesise the results of included sources as this method lacks formal methodological quality assessment and this review included grey literature. Basic frequency counts were employed to describe the results (Peters et al. 2020) while content analysis was used for the qualitative data.

### Ethical considerations

Ethical approval was obtained from the University of Cape Town, Faculty of Health Sciences, Human Research Ethics Committee (HREC Ref: 721/2020).

## Results

Publication year ranged from 2006 to 2020, with a near even split between grey literature (*n* = 16) and primary research (*n* = 15) literature. Of the primary research, there were predominantly mixed study designs (*n* = 7), followed by qualitative (*n* = 5) and then quantitative designs (*n* = 3). All were from high-income countries (HICs), the majority from United Kingdom, or Britain, and Ireland (*n* = 18), followed by New Zealand (*n* = 4), United States (*n* = 3), Australia (*n* = 3), France (*n* = 1), Japan (*n* = 1) and Norway (*n* = 1). Table 1 and Table 2, respectively, lists the primary research and grey literature evidence sources.

**TABLE 1:** Primary research evidence sources.

Authors (year)	Aim of study	Type	Country	Study design
Chan (2008)	To examine the profile of respite services and explore service providers’ views on factors influencing use of respite service and service delivery in NSW	Research article	Australia	Mixed
Cramer and Carlin (2008)	To investigate the state of the current service provision of family-based short breaks in the UK	Research article	UK	Mixed
Holmes, McDermid and Sempik (2010)	To determine costs incurred by Children’s Services Departments by providing short breaks to children with disabilities and their families	Research report	UK	Mixed
Kelly, Craig and McConkey (2020)	To track home-based support services and overnight stays in Ireland, identify associated indicators in the provision of family support services and any changes in the service in the last 10 years	Research article	Ireland	Quantitative
LoGiudice et al. (2012)	To explore development and implementation of a locally designed and culturally appropriate community service care model for older people with disabilities and people with mental health problems in remote Aboriginal Australia	Research article	Australia	Mixed
McClean, Grey and McCracken (2007)	To evaluate the implementation of PBS for five individuals with the most severe challenging behaviours resident within a county in Ireland	Research article	Ireland	Mixed
McConkey, Gent and Scowcroft (2011)	To use a multiinformant approach to document the essential features of a successful short break and community support service by Action for Children in three UK cities	Research article	UK	Qualitative
McConkey, Gent and Scowcroft (2013)	To provide evidence of functioning and effectiveness of a short break service by identifying how three services were perceived to meet the needs of families whose children are severely challenging	Research article	UK	Qualitative
Merriman and Canavan (2007)	To investigate best practice regarding respite care for persons with ID and autism	Research report	Ireland	Qualitative
Nicholson et al. (2019)	To determine differences in QOL across three types of respite care for people with mild to moderate ID	Research article	Ireland	Quantitative
Nishigaki et al. (2017)	To elucidate factors related to desire to use social services and the actual use of respite care services by primary caregivers of children with SMID	Research article	Japan	Quantitative
Roos and Søndenaa (2020)	To explore collaboration process between parents and employees and identify factors that improve the transition of persons with profound ID from home to independent living	Research article	Norway	Qualitative
Southby (2017)	To describe actual or perceived barriers to availing non-residential respite for adults with ID and/or autism with moderate to complex needs	Research article	UK	Mixed
Spooner (2020)	To investigate the ways in which care is understood and provided, and the influence of care on the social worlds of young adults with complex needs in New Zealand	Thesis	New Zealand	Mixed
Stalker and Moscardini (2012)	To inform the work of Scotland’s Commissioner for Children and Young People from 2012 to 2016, in relation to children with disability and young people identified as a priority group and with attention on the issue of social inclusion	Research report	UK	Qualitative

Note: Please see the article Abrahams, T. & Kleintjes, S., 2023, ‘Respite care models and practices for persons with intellectual disability: A scoping review’, *African Journal of Disability* 12(0), a1115. https://doi.org/10.4102/ajod.v12i0.1115 for complete reference list.

ID, intellectual disabilities; PBS, positive behaviour support; NSW, New South Wales; QOL, quality of life; SMID, severe motor and intellectual disabilities; UK, United Kingdom or Britain (includes England, Scotland, Wales and Northern Ireland).

**TABLE 2:** Grey literature evidence sources.

Authors (Year)	Topic	Type	Country
ARCH National Respite Network (2015)	Respite consumer guide for family caregivers	Consumer guide	US
Batata et al. (2017)	Propose and simulate the use of a model to provide and design respite services for the caregivers which considers quality, need and cost	Conference proceeding	France
Bigham, Cunningham and Johnston (2017)	How a school of nursing and faith communities can partner to provide respite, and benefits to stakeholders	Opinion piece	US
Chan et al. (2012)	A shared understanding on respite and a framework to move towards integrated service which includes a continuum of services, and gives families control over service use	Review	Australia
Department of Health (2007)	Support for commissioners to develop local services for people with LD whose behaviour presents a significant challenge	Government report	UK
Department of Treasury (2007)	How to improve outcomes for children with disability, young people, and support their families, and action taken in priority areas, i.e. access and empowerment, responsive services and timely support, and improving quality and capacity	Government review	UK
Dilks-Hopper et al. (2019)	An update on the Ealing Intensive Therapeutic and Short Break Service 5 years after implementation	Discussion Paper	UK
Goodhead and McDonald (2007)	Investigation into significance and impacts of informal caregiving	Government report	New Zealand
Hanrahan (2010)	Survey on family-based short breaks services for children and adults with ID and other disabilities in Ireland	Network report	Ireland
Kiernan (2019)	Comment on The Ealing Intensive Therapeutic and Short Break Services research article and service	Commentary	UK
Ministry of Health (2018)	Engagement with children and young people with disabilities on their perspectives of respite to improve services and inform development of a respite outcomes evaluation framework	Government report	New Zealand
National Advisory Committee on Health and Disability (2010)	Recommendations on how to better support and provide services for informal carers	Government report	New Zealand
NHS England (2017)	Provide guidance for Transforming Care Partnerships in commissioning support and services for children and young people with LD, autism or both and supplement Building the Right Support and the National Service Model	Government report	UK
NHS England (2017)	Resource for health and social care commissioners to develop service specifications to support implementation of the national service model for people with a LD and/or autism who display BTC, including a mental health condition	Government report	UK
Openden et al. (2006)	Steps for identifying potential respite providers via development	Opinion piece	US
Staley (2008)	Information about innovative practices in providing short breaks	Guide	UK

Note: Please see the article Abrahams, T. & Kleintjes, S., 2023, ‘Respite care models and practices for persons with intellectual disability: A scoping review’, *African Journal of Disability* 12(0), a1115. https://doi.org/10.4102/ajod.v12i0.1115 for complete reference list.

BTC, behaviours that challenge; LD, learning disabilities; ID, intellectual disabilities; US, United States; UK, United Kingdom or Britain (includes England, Scotland, Wales and Northern Ireland).

### Characteristics of service models for respite care for persons with intellectual disability

#### Care recipients and caregivers

Respite care services catered for all ages groups (*n* = 11) or for children (*n* = 10) or adults (*n* = 9) separately. One did not specify this detail. More than half (*n* = 19) did not specify severity of ID. Behaviours that challenge (BTC) was mentioned in about half the articles (*n* = 17). Predominately family and parents accessed services.

#### Purpose

Caregiver respite was the sole purpose for almost a quarter (*n* = 8) of the services, while more than half the services had multiple purposes for respite (*n* = 21). Purposes related to caregivers included respite (ARCH National Respite Network 2015; Merriman & Canavan 2007; Southby 2017; Spooner 2020), stress alleviation and burden reduction (Chan 2008) and support (Kelly et al. 2020; McConkey et al. 2011). Purposes for care-recipients included respite (Department of Health 2007; Holmes et al. 2010; McConkey et al. 2011; Merriman & Canavan 2007; Southby 2017), a break from daily routine (Kiernan 2019), skills and independence development (Chan 2008; Southby 2017; Spooner 2020), social inclusion (Department of Treasury 2007; McConkey et al. 2011, 2013; Southby 2017), a place of safety (McClean et al. 2007), prevention of institutionalisation, admission, placement breakdown or out-of-area placement (Dilks-Hopper et al. 2019; National Health Services 2017b), and access to enjoyable, stimulating, constructive and positive activities (McConkey et al. 2011). Purposes for other recipients included family respite (Bigham et al. 2017; Department of Health 2007; Department of Treasury 2007; Holmes et al. 2010; McConkey et al. 2011), family skills development (McConkey et al. 2011) and student learning opportunities (Bigham et al. 2017).

#### Criteria and service terms

Less than a third (*n* = 9) indicated inclusion criteria and fewer indicated exclusion criteria (*n* = 4) to access the service. Under half the articles (*n* = 13) used the term *respite* or *respite care* exclusively while the term *short break* was used exclusively in far fewer (*n* = 5).

#### Types of respite care offered

Both in- and out-of-home respite care was offered (*n* = 24). The most frequently mentioned in-home respite care was provided by formal carers or workers in the home (ARCH National Respite Network 2015; Cramer & Carlin 2008; Department of Treasury 2007; Dilks-Hopper et al. 2019; Goodhead & McDonald 2007; Holmes et al. 2010; Kelly et al. 2020; Merriman & Canavan 2007; Ministry of Health 2018; Nishigaki et al. 2017; Roos & Søndenaa 2020; Southby 2017). Specialist or professional support in the home was also offered where required (ARCH National Respite Network 2015; Holmes et al. 2010; Nishigaki et al. 2017). Other examples of in-home respite care included emergency care in the home (Chan 2008), home help and home care (Department of Treasury 2007; Goodhead & McDonald 2007; LoGiudice et al. 2012; Nishigaki et al. 2017).

A variety of out-of-home respite care was reported. Table 3 provides examples and definitions of some of these. The most frequently reported location of out-of-home respite care was residential homes or care facilities (ARCH National Respite Network 2015; Chan 2008; Department of Health 2007; Department of Treasury 2007; Goodhead & McDonald 2007; Kelly et al. 2020; NHS England 2017a, 2017b; Nicholson et al. 2019; Roos & Søndenaa 2020; Southby 2017; Staley 2008; Stalker & Moscardini 2012). This was followed by day-care centres (Cramer & Carlin 2008; Kelly et al. 2020; Merriman & Canavan 2007; NHS England 2017b; Nishigaki et al. 2017) and hospitals (ARCH National Respite Network 2015; Batata et al. 2017; Chan 2008; NHS England 2017b; Nishigaki et al. 2017). Out-of-home respite also included access to community activities (Cramer & Carlin 2008; McConkey et al. 2011, 2013; National Advisory Committee on Health and Disability 2010; Staley 2008), recreational and leisure activities (LoGiudice et al. 2012; Roos & Søndenaa 2020; Southby 2017), creative activities (LoGiudice et al. 2012), and social activities (Nicholson et al. 2019).

**TABLE 3:** Examples of out-of-home respite care.

Type of out-home respite	Definition
Shared care families	Volunteer caregiver couples have care recipient in their home (Goodhead & McDonald 2007)
Host-family based services	Host families, contract family schemes and home-sharing services that provide family-based accommodation (Hanrahan 2010)
Overnight family based	Foster carers offer overnight break in their homes (Holmes et al. 2010)
Supported access	Support to access universal or targeted services including special equipment or training for service or accompanying child to service (Holmes et al. 2010)
Weekend club	Group activities held over the weekend including supported sports activities, play activities and trips (Holmes et al. 2010)
School holiday activities	Activities provided during school holidays including family fun days, supported sports and crafts, trips to leisure parks and zoos (Holmes et al. 2010)
Specialist holidays	Holiday activities including active holiday breaks and support for family holidays (Holmes et al. 2010)
Under fives groups	Two-hour breaks for parents while children take part in activities adapted to their individual needs (Staley 2008)
School holiday play schemes for children with complex health needs	Specialist groups provide one-to-one support (nursing care) for children who need support to use play facilities, 12 children per day for 24 days per year for a 6-h session (Staley 2008)
Transition summer holiday club	For young people making transition to adulthood, four staff members support them to attend chosen activities which are similar to those of aged peers, with a focus on their safety and peace of mind for carers. Activities include swimming, bowling, picnics, cinema, pubs and restaurants (Staley 2008)
Short break house	Homelike accommodation in which four to five children stay overnight in their own bedroom and share domestic-style living and dining facilities. Buildings adapted in terms of bathrooms and security features. Generous garden and outdoor play areas. Activities in the community (McConkey et al. 2011)

Note: Please see the article Abrahams, T. & Kleintjes, S., 2023, ‘Respite care models and practices for persons with intellectual disability: A scoping review’, *African Journal of Disability* 12(0), a1115. https://doi.org/10.4102/ajod.v12i0.1115 for complete reference list.

#### Duration and scheduling

Many (*n* = 21) offered overnight and day services. Almost half (*n* = 14) offered a combination of short-, medium- and, in fewer instances, long-term stays. Services were offered on weekdays, weekends and holidays. Half (*n* = 16) offered emergency and planned use, with half of those (*n* = 8) offering scheduling flexibility.

#### Package of care

About half (*n* = 17) indicated respite care was part of a package of care, while only a few (*n* = 2) offered respite care exclusively. Over a third (*n* = 12) did not specify this detail. Examples of other package components included medical and allied healthcare (Dilks-Hopper et al. 2019; Merriman & Canavan 2007), assessment, treatment, training, support, transition coordination and crisis response (National Health Services 2017b), regular reviews (Department of Health 2007), educational services (Dilks-Hopper et al. 2019), Positive Behaviour Support (PBS) programmes (Kiernan 2019), home visits (Holmes et al. 2010) and respite funding (Spooner 2020). Over a third (*n* = 12) provided a combination of activities, including recreational, leisure, educational, social and skills development activities but only a few (*n* = 5) specified that they offered activity choices to care-recipients with ID.

#### Service providers

The state was the key provider in most sources (*n* = 25). Most (*n* = 24) providers were formal (paid) providers, with a few services (*n* = 5) using a combination of informal (unpaid) and formal providers. Approximately two thirds (*n* = 20) addressed staffing quality, which focused on experience, skills, training, qualification, supervision, cultural sensitivity, staff support, inclusion of experts by experience, competency assessment, trust, rapport, relationships with users, retention, continuity, remuneration and values and attitudes (e.g. Department of Health 2007; Goodhead & MacDonald 2007; McConkey et al. 2013; Ministry of Health 2018; National Health Services 2017a, 2017b; Southby 2017; Spooner 2020).

#### Funding

Slightly more than half indicated state funding (*n* = 17), with very few (*n* = 3) indicating access to special and innovation funding. Over half (*n* = 18) discussed service cost-effectiveness.

#### Responsiveness to need

Half (*n* = 16) based the respite care service on the needs of caregivers, family and the care-recipient, rather than only the needs of the caregiver. The range of needs assessment approaches included the biopsychosocial approach (National Health Services 2017b), person-centred approach (Department of Health 2007), risk taking assessments (Hanrahan 2010), functional assessments and direct observation (Dilks-Hopper et al. 2019). These looked at, for example, family needs, behaviour, recipient needs and goals (Bigham et al. 2017), caregiver burnout and exhaustion (Batata et al. 2017), and family ability to provide support (McConkey et al. 2011). Assessments were performed by varied stakeholders such as individual professionals, multidisciplinary teams and panels, which assess needs and resource use (Hanrahan 2010; Holmes et al. 2010; McConkey et al. 2011).

### Standards, best-practices, guidelines, service delivery frameworks and principles for providing respite care to persons with intellectual disability

Two thirds of the sources mentioned principles (*n* = 21) and about half reported on best practices (*n* = 16), while service frameworks (*n* = 9), guidelines (*n* = 8), standards (*n* = 6) and norms (*n* = 1) were mentioned less frequently. Table 4 summarises the data extracted on principles, best practice, service delivery frameworks, and guidelines for respite care. Standards mentioned included National Minimum Standards for Children’s Homes (Holmes et al. 2010), Supporting People with Profound and Multiple Learning Disabilities Core & Essential Service Standards (Spooner 2020) and NICE Guidelines for challenging behaviour and learning disabilities and autism (NHS England 2017b). Care Standards in the UK also offer a means to improve standards of practice and a way to evaluate services (Cramer & Carlin 2008), while National Minimum Standards are needed (Hanrahan 2010). Standards can also aid consistency of procedures in services for those with BTC (McConkey et al. 2011). Only Roos and Søndenaa (2020) reported on norms, specifically guidelines on staffing norms for services for children with profound ID.

**TABLE 4:** Principles, best practice, service delivery frameworks and guidelines.

Authors (Year)	Principles	Best practice	Service delivery framework	Guidelines
ARCH National Respite Network (2015)	Implicit principles for caregivers accessing respite care e.g. consider earlier than needed	-	-	-
Batata et al. (2017)	-	-	Types of, access to, assessment for and availability of respite beds	-
Bigham et al. (2017)	Quality respite care e.g. affordable and evaluation to inform improvement	-	-	-
Chan (2008)	Simplified access to respite services. Consumer driven services. Prediction of use and non-use to aid service planning, resource allocation. Shift from crisis to prevention.	Flexible respite options and funding models	-	-
Chan et al. (2012)	Shared understandings, integration and person-centred funding	Flexible respite options and flexible funding models are key features of an ideal respite service	-	
Cramer and Carlin (2008)	Consultation with service users	-	-	-
Department of Health (2007)	-	Practices for short break services for those with ID and BTC e.g. tailored to individual need, available during crises	-	-
Department of Treasury (2007)	Principles to inform and improve respite care service provision e.g. *Every Child Matters* in the UK	Respite care best practice e.g. innovate and flexible provision		
Dilks-Hopper et al. (2019)	-	Best practice service models	PBS as an operations framework which includes short breaks and intervention	Guidelines that include respite care e.g. NICE guidelines
Goodhead and MacDonald (2007)	Principles of good quality respite care e.g. planned, provides feedback to caregiver and training and/or supervision to formal caregiver	-	-	-
Hanrahan (2010)	-	Network that develops good practice	-	Guidelines for assessment of short break hosts
Holmes et al. (2010)	Cost considerations e.g. when calculating unit costs for breaks consider that costs vary according to needs, social care activity, service type and overhead calculations	Best practice service models e.g. family choice and use of direct payments	-	-
Kelly et al. (2020)	A wider variety of family support options, a national database of service usage to identify issues needing attention, attention to the needs of persons with ID	-	Geographical and organisational division and responsibility e.g. CHO must commission services in line with policies	-
Kiernan (2019)		Best practice service models	-	Guidelines that include respite care e.g. NICE guidelines
LoGiudice et al. (2012)	Principles of success for remote community health programmes e.g. cultural comfort and adequate funding	Good practice when delivering services to aboriginal people e.g. community participation and cultural protection	-	-
McClean et al. (2007)	Principles for intervention for people with ID and BTC e.g. should include adapted respite facilities with psychiatric support	-	-	-
McConkey et al. (2011)	-	-	Key service features for those with BTC e.g. for short breaks compatible groupings should be accommodated	Standalone short break guidelines. Recommendations to improve existing guidelines e.g. distinct specialist services needed for families with complex needs beyond breaks
McConkey et al. (2013)	Principles for intervention for those with ID and BTC e.g. short breaks can prevent placement when there is trusting relationships between families and agencies	-	-	-
Merriman and Canavan (2007)	Respite care principles e.g. provided on a rights basis and designed in consultation with families	Respite care best practice principles e.g. person- and family-centred and part of a system of supports	How services should be delivered, oriented, evaluated and the community involved e.g. a single point of entry to services	-
Ministry of Health (2018)	-	Best practice to engage children about respite services e.g. adapt method to target group and allow more time	-	Standalone respite care guidelines from different groups e.g. for children with autism to have support workers who understand autism. Recommendations advanced that could inform improvements to existing guidelines
National Advisory Committee on Health and Disability (2010)	Service contracts where services observe principles such as ensuring high QOL for people with disabilities	-	-	-
NHS England (2017b)	Principles for intervention for those with ID with BTC e.g. transformation underpinned by values in which individual and QOL are centre	Respite care best practice e.g. close to people’s homes and early detection of need	PBS can be an operations framework and approach	Use of existing guidelines that include standards and inform services
NHS England (2017b)	-	Best practice related to short breaks e.g. a full range of specialist respite services and age appropriate residential and non-residential options	-	-
Nicholson et al. (2019)	Personalisation, i.e. looking at the person rather than the service	Personalised service	-	-
Nishigaki et al. (2017)	Principles to encourage respite care use e.g. caregiver should see evidence of child enjoying respite	-	-	-
Openden et al. (2006)	Development of lists by educational institutions to develop support for families in the local community	-	How services can be setup e.g. recruiting students for respite care	-
Roos and Søndenaa (2020)	-	-	-	Guidelines that include respite care e.g. provision of information on respite care
Southby (2017)	Principles for non-residential respite e.g. use as adjunct to residential respite care and consider timing	-	-	-
Staley (2008)	Principles advanced by children and caregivers e.g. children do not necessarily want breaks without their families and parents want practical help	Respite care best practice e.g. responsive to family needs and offers stimulating and educational activities. Examples of best practice service models	Mandates for service provision e.g. local authorities must provide short breaks for children with disability	-
Stalker and Moscardini (2012)	Guiding principles for short breaks e.g. inclusive and broader definition	Short breaks best practice e.g. must be positive experience for parent and child	Existing frameworks that can be used e.g. GIRFEC	Standalone respite care guidelines e.g. minimum entitlement to short breaks and short breaks should enhance QOL

Note: Please see the article Abrahams, T. & Kleintjes, S., 2023, ‘Respite care models and practices for persons with intellectual disability: A scoping review’, *African Journal of Disability* 12(0), a1115. https://doi.org/10.4102/ajod.v12i0.1115 for complete reference list.

BTC, behaviours that challenge; CHO, Community Health Organisation; GIRFEC, Getting it Right for Every Child; ID, intellectual disabilities; NICE, National Institute for Clinical Excellence; PBS, positive behaviour support; QOL, quality of life; UK, United Kingdom.

Under a quarter (*n* = 5) mentioned culture in guiding service offerings. For example, LoGiudice et al. (2012) referred to cultural protection and comfort, National Health Services (2017a) referred to cultural sensitivity, Goodhead and McDonald (2007) referred to cultural safety while Nishigaki et al. (2017) suggested avenues to address caregiver guilt, which can be elicited by cultural norms when services are accessed. National Advisory Committee on Health and Disability (2010) found respite care was not viewed as a substitute for *whaānau* or family-provided care in New Zealand.

### Respite care evaluation and impact

Services were evaluated in slightly more than half (*n* = 17) of the sources. Service evaluation methods included caregiver interviews (Merriman & Canavan 2007), satisfaction and evaluation surveys (Chan 2008), service audits and developmental evaluations (National Advisory Committee on Health and Disability 2010), independent evaluations (LoGiudice et al. 2012), use of outcomes frameworks (National Health Services 2017b), teacher assisted interviews and online surveys (Ministry of Health 2018), a national ID database (Kelly et al. 2020), psychometric assessment, medication review, service review and revenue costs (McClean et al. 2007). One source described formal 6-monthly service-led, multiagency home-based reviews (McConkey et al. 2011). Few studies (*n* = 3) used specific assessment tools. Positive outcomes of respite care for parents were found using the Parenting Stress Scale (Bigham et al. 2017) and for care recipients with BTC using the Quality-of-Life Questionnaire (McClean et al. 2007). Nicholson et al. (2019) found no difference on self-report but differences in proxy measures of quality of life using the Self-Reported Quality of Life Scale and the INICO FEAPS to measure quality of life for persons with mild to moderate ID. Identified impacts included continued support and delayed out-of-home placement (Dilks-Hopper et al. 2019) and increased service provision, usage, improved service quality and access (LoGiudice et al. 2012). Evaluation also demonstrated benefits to a range of recipients including caregivers, care-recipients with ID and families (McConkey et al. 2011, 2013) and respite providers (Bigham et al. 2017; Openden et al. 2006).

## Discussion

The review provides rich information on respite care models and practices albeit only from HICs. The existence of these services coupled with the principles that inform these reinforces the importance of offering good quality respite care to caregivers of those with ID, who have particular support needs (Lee, Burke & Perkins 2022; Lunsky et al. 2014), especially because informal caregivers represent a significant population of those who care for persons with ID in the community (Lunsky et al. 2014). The review results suggest that respite care is one important tool to actualise informal caregivers’ right to support, not only through its intended purposes and impacts but also through empowered provision, which the principles and practices espouse. While states rely on informal caregivers to care for citizens with disability, over-reliance on informal caregivers can have detrimental effects on caregivers and care-recipients (WHO 2011). Service provisions should be aligned to enforce the right to respite care and other rights such as the right to participation as elaborated in the CRPD, as has been conceptualised, for example, in respite care services in New Zealand (Ministry of Health 2017). In contexts where this is less developed, great care should be taken in policy guidelines, service development designs and resourcing to guard against simplistic understanding of respite care as a mere break for family caregivers (Chesson 2001), and instead to promote understanding that it is in service of the health and well-being of multiple stakeholders (Aldersey et al. 2016; Welch et al. 2012; Whitmore 2017).

The results provide clarity on how respite care services are delivered. Respite care is a primary service offering, not an incidental benefit of another service, which reinforces the importance of explicitly offering this kind of this service (Neece & Lima 2016). That most of the formal services are focused on both family caregivers and care-recipients, and have multiple purposes beyond merely offering a caregiver break, is likely impacted by contemporaneous definitions of respite care. These definitions highlight that respite care should benefit caregivers, care-recipients with ID and families (Kirk & Kagan 2015; Robertson et al. 2011; Whitmore 2017). The importance of a lifespan approach to respite care provision is documented (Kirk & Kagan 2015; Remedios et al. 2015) and echoed in the findings of this review, which included an even spread of data on respite care services for both children and adults. Children with ID with inadequately supported caregivers are, for example, more likely than their peers without disability to be placed in out-of-home care and residential care where their attachment, social and emotional development can be negatively impacted and where they are more at risk for maltreatment (Shannon, Wilson & Blythe 2023). The literature also suggests that respite care services for adults with ID need further attention because of, for example, compound caring where older caregivers care for older adults with ID (Lee et al. 2022). The severity of ID was not often specified, possibly because the services are based on individual support needs, not intelligence quotients, in accordance with best practices and current definitions of ID. Inclusion and exclusion criteria were not often specified; however, service users and referring providers need access to this information to know what services are accessible. This omission may result because clear policies exist in HICs around respite care access to guide referral processes (Mencap 2018; Ministry of Health 2017). The range of in- and out-of-home offerings provide diverse and developmentally appropriate activities and elicit exciting possibilities for what could constitute respite care in different settings. Murphy, Begley and Doyle (2021) reinforce the need to offer a range of responsive respite care services, while Guerin et al. (2021) highlight the importance of alternative models of respite care. A focus on flexibility in duration, timing, and frequency of use, with emphasis on the need for crisis responses on a 24-h basis, especially for persons with ID and BTC seems underpinned by best practice and is supported in the literature (McCombe et al. 2022). Long-term stays were minimally specified in line with the right to community living and deinstitutionalisation for persons with ID (Mansell 2006).

The review highlights that respite care as a component of a package of care should be determined by need. The needs of both parties are considered; however, caregiver needs still dominate in the literature (Nankervis et al. 2011). This is possibly exacerbated by service provider’s communication and training barriers to directly assess care-recipient’s needs (Kittelsaa 2004). The results show that evaluations to identify service provision impacts are undertaken using multiple perspectives and varied tools; however, a specific tool to measure respite care benefits such as that developed by Otsuki, Fukui and Sakaguchi (2020) may prove useful to provide quantitative evidence, which is lacking in this area (IASSIDD 2014). The implication of the review for service delivery considerations is as follows: that utilised definitions and terms need to be as contemporaneous and as uniform as possible, there needs to be clear policies that provide information about service access, the varied forms of respite care need to be embraced and further developed in different contexts, rights provisions should and can be upheld when providing quality respite care, and packages of care should be offered based on caregiver and care-recipient needs, with special effort to determine the needs of persons with ID.

The review also highlighted a significant sub-population, namely persons with ID and BTC, who require specialist intervention to protect their rights because they are at far greater risk of social exclusion (Bigby et al. 2012). The presence of BTC can have pervasive negative impacts on caregivers and care-recipients (Kiernan et al. 2019). For persons with ID especially, it can result in infringement of their rights to community living when placement breaks down and they are institutionalised in restrictive settings (Reid et al. 2013). Respite care is a necessary care package component for these individuals and their families, as is training, support, intervention and an intersectoral approach to care (McConkey et al. 2011). For example, respite care and skills development may serve as preparation for independent living for older adults with ID and BTC (Tilley et al. 2022). Intervention should be offered as early as possible and based on individual and family needs (Kiernan et al. 2019). Evidence of good practice in HICs with this subset of the ID population can be used and informed by research on how to adapt the model to different settings without losing the essence of what works (Coetzee et al. 2019).

The review showed that HICs’ state support systems play an important role in service provision to families of children with disabilities (Nuri, Batorowicz & Aldersey 2020). States have a pivotal role in funding responsive formal public sector support services and in setting standards and regulating services (WHO 2011). State support enables the use of formal providers by empowering informal caregivers, for example when such caregivers are remunerated for their labour via US Medicaid waivers for people with ID (Friedman & Rizzolo 2016) or enabled to access respite care through Medicaid Home and community-based services (Eskow, Pineles & Summers 2011). State support for respite care may also be mandated by legislation and policy in the countries included the review, which in turn allows respite care budget allocations to aid provision. For example, New Zealand’s 2017–2022 Respite Strategy is founded on numerous legislative instruments and makes provision for varied forms of funding, framing respite as an investment in health and well-being of its citizens (Ministry of Health 2017). Nuri et al. (2020) affirm the importance of policies that make provision for financial support to families who cannot afford the costs of raising children with disabilities in LMICs. Implications of these findings include the need for legislation and policy to support respite care provision and to open budgeting avenues, the latter of which can be supported by research into the cost-effectiveness of contextually appropriate respite care models in LMICs settings.

A significant finding was the lack of published research focused on respite care for persons with ID in LMICs. This mirrors the imbalance of published ID research in general compared with HICs (McKenzie et al. 2013). A similar picture exists for research on support for families of children with disabilities in LMICs (Nuri et al. 2020). While some literature touches on respite care in LMICs (e.g. Aldersey et al. 2016) it does not offer specific information on this kind of service provision. The lack of published research on respite care for those with ID in LMICs may result from services not being formally documented in research. A reason for this may be that mental health professionals in LMICs and by extension other professionals involved in ID care, have tended to respond to caregiver intervention needs with innovative approaches but without research to inform policy (Murthy 2016). Research funding for ID also takes place in a competitive environment where other research priorities take precedence (Holland 2010), and funds may not as yet be available to invest in such research. An implication is that published research on respite care for persons with ID in LMICs needs to be encouraged and funded.

The lack of published research on respite care in LMICs may also suggest a gap in formal respite care services. Nuri et al. (2020) found that families still rely more on informal support, inclusive of respite care from family in LMICs, in line with earlier findings that formal disability support services are more common in HICs (WHO 2011). In Africa, for example, formal respite care is not as readily available, with only 14% of African countries offering this service (WHO 2007), despite 65% of African countries ratifying the CRPD (Lord & Stein 2013). Nuri et al. (2020) argue that the difference in extent of formal supports, including respite care for those with disabilities, between HICs and LMICs results from economic, cultural and social contextual differences where increased poverty, limited health and social care systems, stigma and discrimination and cultural values play a role in LMICs. Some African studies reinforce this argument. For example, poverty presents significant challenges for family caregivers (McNally & Mannan 2013; Mkabile & Swartz 2020) as does stigma and discrimination (Mkabile et al. 2021; Tilahun et al. 2016). If efforts are made to correct the service imbalance in LMICs, the information found in this review could be adapted for use; however, context influences provision (Evans 2013) and the lack of LMIC respite care research means that specific contextual factors and constraints are unknown. Societal level culture, as a contextual factor, needs exploration because the review showed that culture plays a role in services, possibly underpinned by the right to cultural identity in the CRPD (UN 2006). Culture informs access to and respite care use (Durà-Vilà & Hodes 2009; Neely-Barnes & Dia 2008; Van Den Mark et al. 2019). Culture also influences what is understood as respite (Dysart-Gale 2007), what constitutes acceptable approaches to and who is responsible for care (Murthy 2016) as well as impacts on stigma experienced by caregivers (Hussain & Raihan 2022). It also remains to be seen how the identified best practices, which are funded to permit individually focused service offerings in many instances, may need to be adapted to align with the values, practices and funding envelopes of more collectivist, culturally different settings. For example, a best practice observed in this review is tailoring intervention to individual need. This might conflict with the needs of the family and community, considered equally important from an interdependence perspective or when family and service definitions of BTC do not resonate with each other (Hatton et al. 2010). Understanding that caregivers value and need respite care (Lunsky et al. 2014; Nuri et al. 2020), an implication is that researchers should study the extent and provision of respite care services offered in LMICs. Researchers and service providers also need to establish if existing HICs respite care offerings, practices and principles can meet the needs of service users in LMICs contexts, and if not, how to setup services to run responsively, appropriately, cost-effectively and sustainably (Coetzee et al. 2019).

### Limitations

While the search was as exhaustive as possible within the constraints of the scope of the study, there may have been further unpublished grey literature on respite care in LMICs. A review of websites of organisations who offer respite care or inclusion of languages other than English may have revealed more literature on respite care in LMICs. While the search may not have been exhaustive enough to ensure all country reports were included, those that were found were from the hand-search of the initially included sources, which met the inclusion criteria, and were thus relevant. That the protocol was not registered was a limitation; however, reviews that commence with a detailed protocol, registered or not, can meet the requirement of transparency and reporting bias (Khalil et al. 2021).

## Conclusion

The review has shed light on how respite care services are offered, specifically in HICs. The existence of a knowledge base of respite care principles and practices that draw on a rights perspective can be harnessed to ensure good quality respite care services are offered in other settings. The lack of respite care information for LMICs, however means there is a gap in understanding the full extent and nature of respite care in these settings. This should be addressed to ensure development and provision of contextually appropriate ID respite care, which is responsive, sustainable and effective. Respite care research in LMICs can bridge the identified gap and aid advocacy efforts for respite care policy and practice.
